# The utility of long non-coding RNAs in chronic obstructive pulmonary disease: a comprehensive analysis

**DOI:** 10.1186/s12890-023-02635-w

**Published:** 2023-09-11

**Authors:** Qi Lin, Chaofeng Zhang, Huixin Weng, Yating Lin, Yucang Lin, Zhipeng Ruan

**Affiliations:** 1 Department of Pharmacy, The Affiliated Hospital of Putian University, Putian, Fujian Province China; 2 Pharmaceutical and Medical Technology College, Putian University, Putian, Fujian Province China; 3 Department of Hematology and Rheumatology, The Affiliated Hospital of Putian University, Putian, Fujian Province China; 4 Key Laboratory of Translational Tumor Medicine in Fujian Province, Putian University, Putian, Fujian Province China; 5 Department of Information, The Affiliated Hospital of Putian University, Putian, Fujian Province China

**Keywords:** Chronic obstructive Pulmonary Disease, Long non-coding RNA, Bibliometric analysis, Diagnostic test accuracy, Meta-analysis, Bioinformatics analysis

## Abstract

**Objectives:**

Chronic obstructive pulmonary disease (COPD) is one of the main causes of morbidity and mortality in the world. However, there are some patients who are not diagnosed early and correctly through routine methods because of inconspicuous or serious symptoms. This study aims to assess the diagnostic role of long non-coding RNA (lncRNA) in COPD.

**Methods:**

We searched literature from electronic databases, after excluding non-COPD literature, the bibliometric analysis was performed, and VOSviewer software was used to represent the data analyzed. Literature evaluating the diagnostic test accuracy of lncRNA for COPD was eligible, and the QUADAS-2 checklist was used to evaluate the quality. The pooled sensitivity (SEN), specificity (SPE), diagnostic odds ratio (DOR), and summary receiver operating characteristic curve (sROC) were used to analyze the overall diagnostic performance. Subgroup and meta-regression analyses were performed to explore the heterogeneity, and a funnel plot was assessed for publication bias. Also, lncRNAs related to COPD were identified and explored for their potential biological function.

**Results:**

An increased annual growth rate of literature on this subject from 2016 focused on COPD, humans, RNA, and lncRNA. The meta-analysis enrolled 17 literature indicated that the SEN, SPE, and DOR differentiating COPD patients from normal controls (NCs) were 0.86 (95% CI [0.80, 0.90]), 0.78 (95% CI [0.67, 0.86]), and 21.59 (95% CI [11.39, 40.91]), respectively. Meanwhile, lncRNAs had the ability to distinguish acute exacerbations of COPD (AECOPD) patients from COPD; the SEN, SPE, and DOR were 0.75 (95% CI [0.62, 0.85]), 0.81 (95% CI [0.71, 0.89]), and 13.02 (95% CI [7.76, 21.85]), respectively. The area under the sROC were calculated to be greater than 0.8 at least. Subgroup and meta-regression analysis showed that the types of specimens and dysregulated lncRNAs might affect the diagnostic accuracy. The funnel plot showed there was a certain publication bias. 41 lncRNAs related to COPD were identified and mainly located in the nucleus and cytoplasm, associated with proliferation, invasion, and prognosis. These lncRNA-binding proteins were involved in the spliceosome, Rap1 signaling pathway, MAPK signaling pathway, and so on.

**Conclusion:**

LncRNA suggests potential diagnostic biomarkers and therapeutic targets for COPD patients.

**Supplementary Information:**

The online version contains supplementary material available at 10.1186/s12890-023-02635-w.

## Introduction

Chronic obstructive pulmonary disease (COPD) is one of the most common chronic lung diseases with chronic bronchitis, emphysema, and incompletely reversible airflow limitations [1–3], which is the third leading cause of mortality worldwide. In China, the prevalence among people aged 40 years or older is about 13.6% [4], and the proportion of COPD patients increases with age [2]. With a focus on disease prevention and health promotion, the Health China Operation (From 2019 to 2030) has called for strengthening early-stage intervention and management of COPD [5]. Over the past decade, increased evidence have indicated that early diagnosis of COPD is essential for easing impact of medical service and reducing the burden on healthcare systems. However, a number of early stage COPD patients undergo misdiagnose [6, 7] because the respiratory symptoms are relatively insidious, the pulmonary function tests are not been cooperated by patients, and the abnormal symptoms that treated with drugs are not checked out [7, 8], leading to treatment and healthcare difficulties.

Several studies have shown that long non-coding RNAs (lncRNA) play an important role in the occurrence and development of many diseases. lncRNA is defined as RNA longer than 200 nt, and its structure is similar to mRNA [9]. At present, many studies have manifested that lncRNA has become a modular biomarker in several diseases [10–12]. Meanwhile, there are many studies that have accessed the diagnostic and prognostic value of lncRNAs in COPD [13, 14]. For example, upregulated expression of the lncRNA *NEAT1* is positively correlated with the The Global Initiative for Chronic Obstructive Lung Disease (GOLD) stage and the levels of inflammatory cytokines tumor necrosis factor (TNF)-a, interleukin (IL)-1β, IL-6, and IL-17 in acute exacerbations of COPD (AECOPD) patients and stable COPD patients, suggesting that *NEAT1* is susceptible to COPD, related to the risk of acute attacks, and is positively related to the severity of the disease and inflammation [13, 15]. LncRNA *MALAT1* expression is higher in AECOPD than in stable COPD and healthy controls, which is also positively correlated with GOLD stage in COPD patients [14, 16]. Generally, lncRNAs might be described as being changed and implicated in the mild and moderate stage of COPD [17], revealing that lncRNAs might be potential diagnostic biomarkers in COPD. However, evidence-based medicine studies of lncRNA in COPD diagnosis are lacking.

This study aims to analyze recent studies about the roles of lncRNAs in the diagnosis of COPD, with a focus on their impact on the clinical progress of COPD and their current clinical application. First, we collected all relevant literature from the electronic database and provided an overview of the enrolled literature. Second, a meta-analysis is conducted to explore the effect of lncRNAs as non-invasive biomarkers. Finally, we also investigated the potential mechanisms by which lncRNAs might be involved in regulation.

## Materials and methods

### Search strategy

This study protocol had been registered in The International Prospective Register of Systematic Reviews (PROSPERO) as CRD42021245500. Two independent authors (LQ and ZC) screened relevant retrospective or prospective published literature following the requirements of the Preferred Reporting Items for Systematic reviews and Meta-Analyses (PRISMA) guidelines for systematic reviews and meta-analysis from English and Chinese electron databases, including Web of Science (WOS), PubMed, Embase, the Cochrane Central Register of Controlled Trials (CENTRAL), China Biology Medicine (SinoMed), China National Knowledge Infrastructure (CNKI), and WangFangData, up to July 31st, 2023. The search terms using subject terms and free words are: “Pulmonary Disease, Chronic Obstructive”, “Lung Diseases, Obstructive”, “Chronic Obstructive Pulmonary Disease”, “COPD”, “emphysema”, “RNA, Long Noncoding”, “lncRNA”, “long ncRNA”, etc. The detail of search strategies are shown in Supplementary Table S1-5.

### Study selection

All screened records were imported into Zotero software (version 6.0), and WPS office software (Kingsoft Office Corp., China) was used to collect data, which included characteristics of eligible studies (e.g., first author’s name, the published date, study region, and index tests), characteristics of participants (e.g., gender, mean age, tested sample, detection method, and the expression of lncRNA), and clinical outcomes conducted for diagnosis. The literature were assessed by two independent authors (LQ and ZC) according to the following inclusion criteria: (1) Published and original studies. (2) Targeted participants were diagnosed with COPD. (3) Including at least one lncRNA tested per study. (4) Primary outcome measures were reported or provided sufficient information to calculate the true positive (TP), false positive (FP), true negative (TN), false negative (FN), sensitivity (SEN), specificity (SPE), and area under the curve (AUC). (5) Language restriction with English and Chinese. Moreover, the exclusion criteria were as follows: (1) Repeated, not original studies. (2) The research data was incomplete. (3) Without assessing diagnostic performance. (4) Studies designed as letters to the editor, study protocols, conference abstracts, case reports, non-human studies, reviews, meta-analyses, editorials, and posters. Duplicate literature were excluded.

### Bibliometric analysis

In order to explore the trend and focus on the literature enrolled, a bibliometric analysis was made. After reviewing the titles and abstracts of screened literature and excluding non-COPD literature, data called “COPD-related data” included literature characteristics (e.g., types, published year, authors, institutions, journals, and countries) and were exported as a bibtex file (Supplementary File S1). Using the bibliometrix package [18] in R programming (version 4.2), the annual growth and the word clouds of published literature were performed. Subsequently, VOSviewer software (version 1.6.19) [19] was used to create a co-occurrence network based on the title, abstract, and keywords of “COPD-related data”.

### Diagnostic test accuracy

For investigating the diagnostic accuracy of lncRNA in COPD, diagnostic test accuracy (DTA) was determined. Data identified as “lncRNA-related data” (Supplementary File S2) were subsequently performed to determine whether the pre-established inclusion and exclusion criteria were satisfied by two independent authors (LQ and ZC). Full-text literatures of “lncRNA-related data” assessed for eligibility, “DTA-related data” were extracted (Supplementary File S3). The Quality Assessment of Diagnostic Accuracy Studies 2 (QUADAS-2) checklist [20] was used to assess the quality of “DTA-related data”, and visualized using the ggplot2 package. All analysis were done using the meta and mada packages [21]. Moreover, DTA would be conducted for pooled SEN, SPE, diagnostic odds ratio (DOR), summary receiver operating characteristic curve (sROC), and calculated Area under the curve (AUC) to analyze the overall diagnostic performance. Heterogeneity was assessed visually by forest plots and analyzed by the Cochran Q test and I^2^. The fixed-effects model was used if there was no significant heterogeneity (I^2^ ≤ 50% or *P* ≥ 0.1); otherwise, the random-effects model was used. Also, we conducted subgroup analysis, sensitivity analysis, and meta-regression to explore the potential sources of heterogeneity in our study. A funnel plot was assessed for publication bias in the enrolled literature. *P* < 0.05 was indicated statistically significant. Additionally, the references of relevant literature were also reviewed to identify any additional studies. Any disagreements of results were solved through discussion or consultation with the other author (RZ).

### Bioinformatic analysis

Next, lncRNAs were identified from “lncRNA-related data” and further assessed for their potential biological function. We attempted to explore the subcellular location and hallmark through the LncSEA platform (http://bio.liclab.net/LncSEA/index.php) [22]. The lncRNA binding proteins were extracted and analyzed through The Database for Annotation, Visualization, and Integrated Discovery (DAVID, https://david.ncifcrf.gov/home.jsp) [23], The functional analysis of the lncRNA binding proteins, including Gene ontology (GO) enrichment and the Kyoto encyclopedia of genes and genomes (KEGG) pathway enrichment [24], was analyzed, and visualization was done using ggplot2 package.

## Results

### Characteristics of enrolled literature

**Fig. 1:**
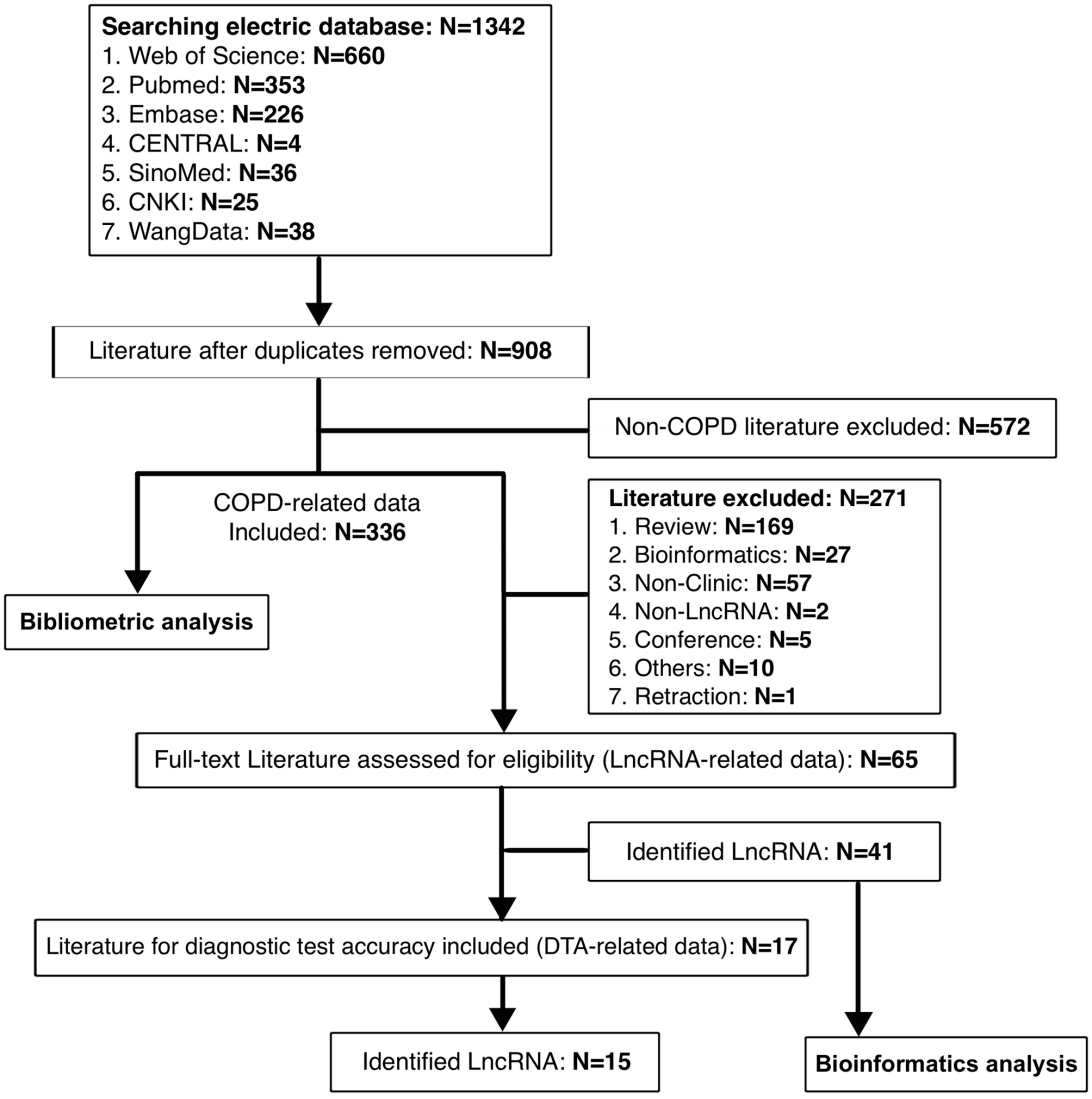
A flow diagram of literature selection

**Fig. 2:**
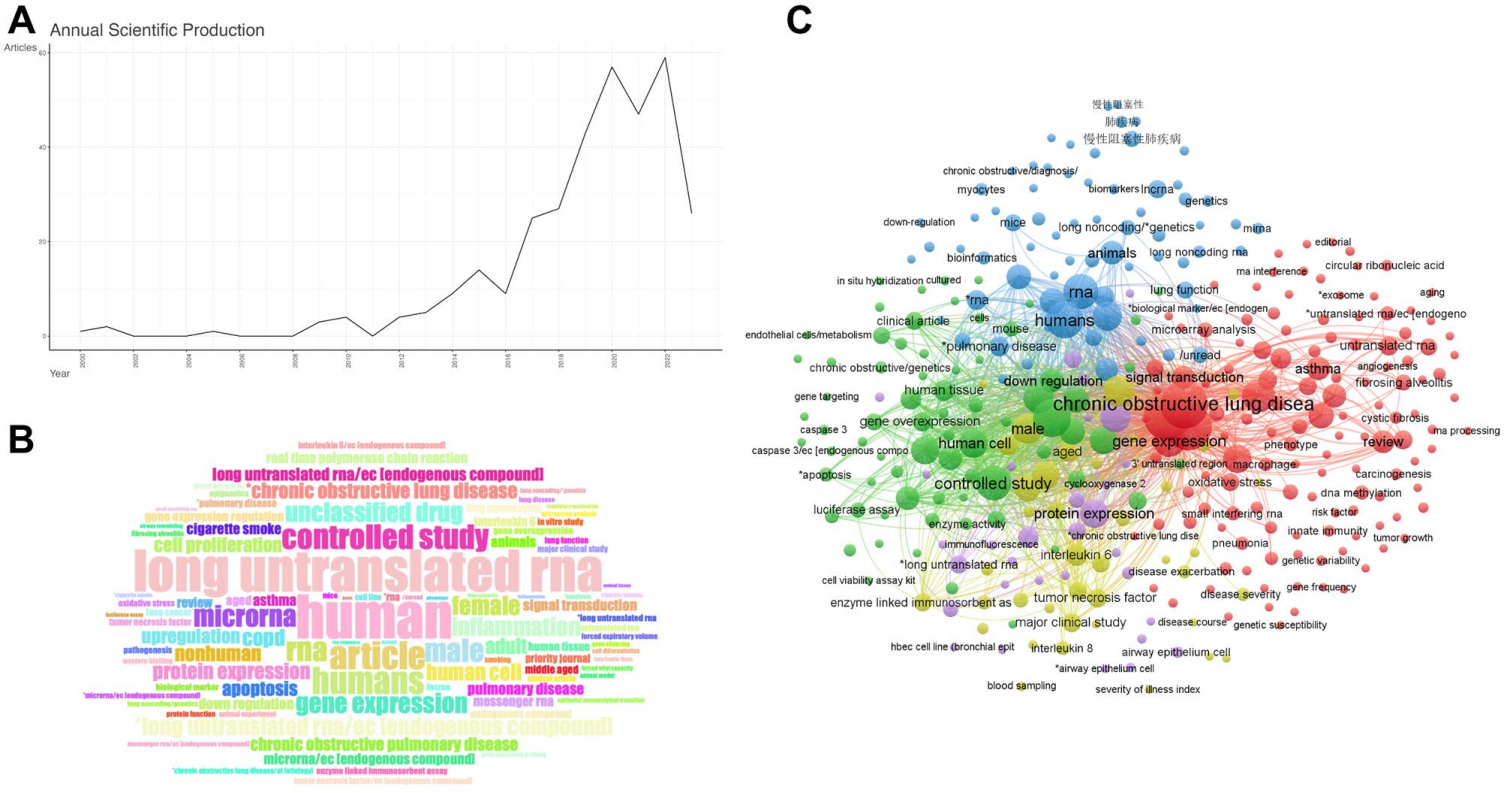
Bibliometric analysis of selected literature. (A) The trend of the numbers of literature per year, (B) Word cloud of selected literature, (C) The co-occurrence network of selected literature

The process of the study is shown in Fig. 1. A total of 1342 literature were enrolled through the comprehensive retrieval. There were 908 literature after removing the duplicates. Next, the titles and abstracts of all studies were screened, and 572 literature were excluded for non-COPD research. The bibliometric analysis was performed using 336 literature (COPD-related data). As shown in Fig. 2, it illustrated the trend in literature published on the subject since 2000, showing that a rapid upward trend was observed in 2016. ‘Chronic obstructive lung disease’, ‘long untranslated RNA’, ‘RNA’, and ‘controlled study’ were identified as the most common words in the identified literature. In the network of co-occurrences of the most frequently used keywords, the terms ‘long untranslated RNA’, ‘Chronic obstructive lung diseaes’, ‘human’, and ‘human cell’ were the most frequently used. After excluded 271 literature, the full texts of 65 literature (lncRNA-related data) were further consideration, and there were 41 lncRNAs identified, including *ANRIL*, *CASC2*, *COPDA1*, *FENDRR*, *GM15621*, *H19*, *HOTAIR*, *ICL*, *IL6-AS1*, *LASI*, *LINC00599*, *LINC00612*, *LINC00987*, *LINC01414*, *LINC00824*, *LincRNA00612*, *LISP1*, *Lnc-IL7R*, *LUCAT1*, *MALAT1*, *MCM3AP*, *MEG3*, *MEG8*, *MIR155HG*, *NEAT1*, *NNT-AS1*, *NR-026690*, *ENST00000447867*, *OIP5-AS1*, *PACER*, *PTPRE-AS1*, *PVT1*, *RNA00882*, *SAL-RNA1*, *SAL-RNA2*, *SAL-RNA3*, *SNHG3*, *SNHG5*, *THRIL*, *TTN-AS1*, *TUG1*.

### Diagnostic performance

**Fig. 3:**
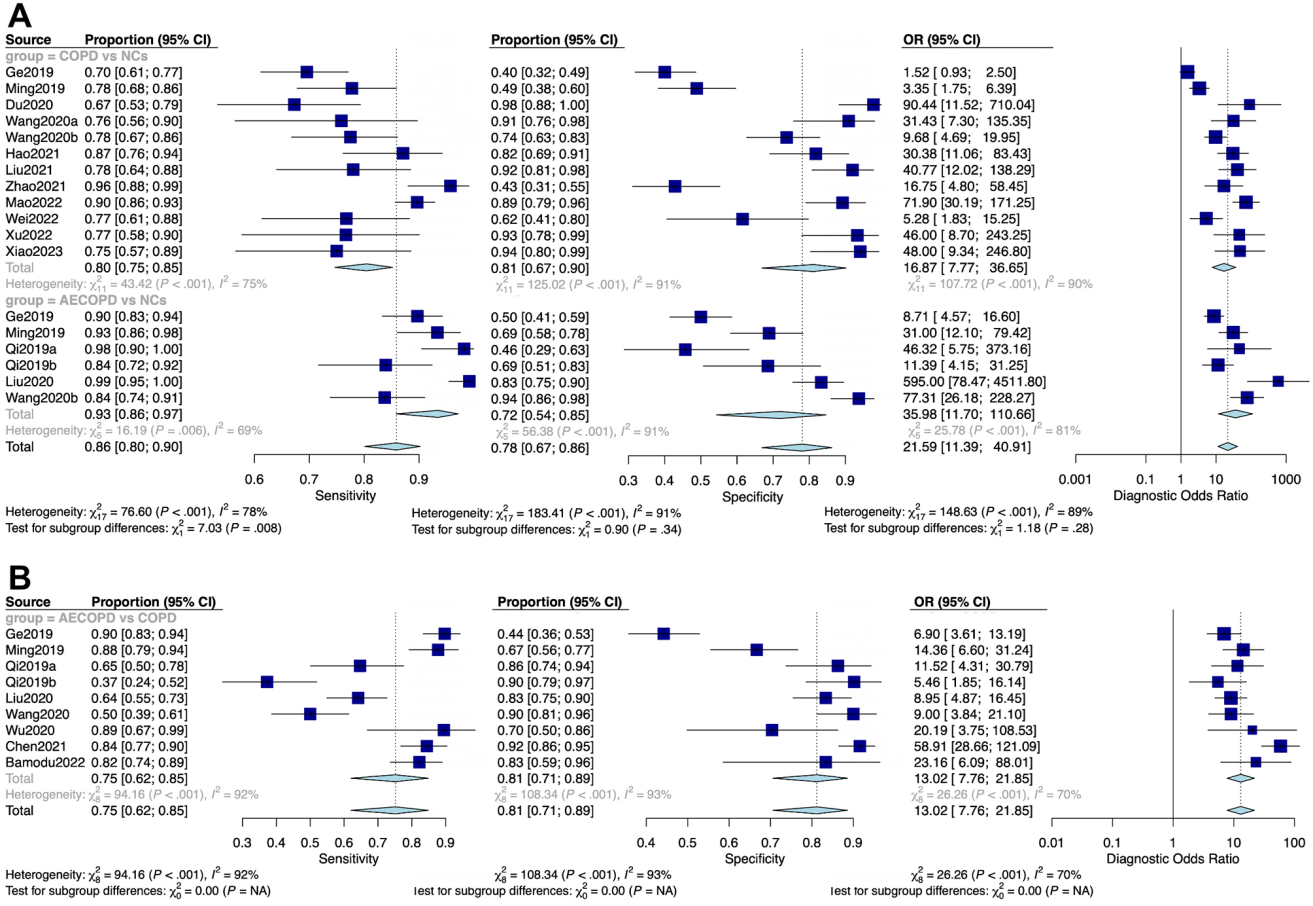
Forest plots for the sensitivity, the specificity and diagnostic odds ratio in meta-analysis. **(A)** COPD vs. NCs, **(B)** AECOPD vs. COPD. AECOPD, acute exacerbations of Chronic obstructive pulmonary disease. COPD, Chronic obstructive pulmonary disease. NC, normal control

**Fig. 4:**
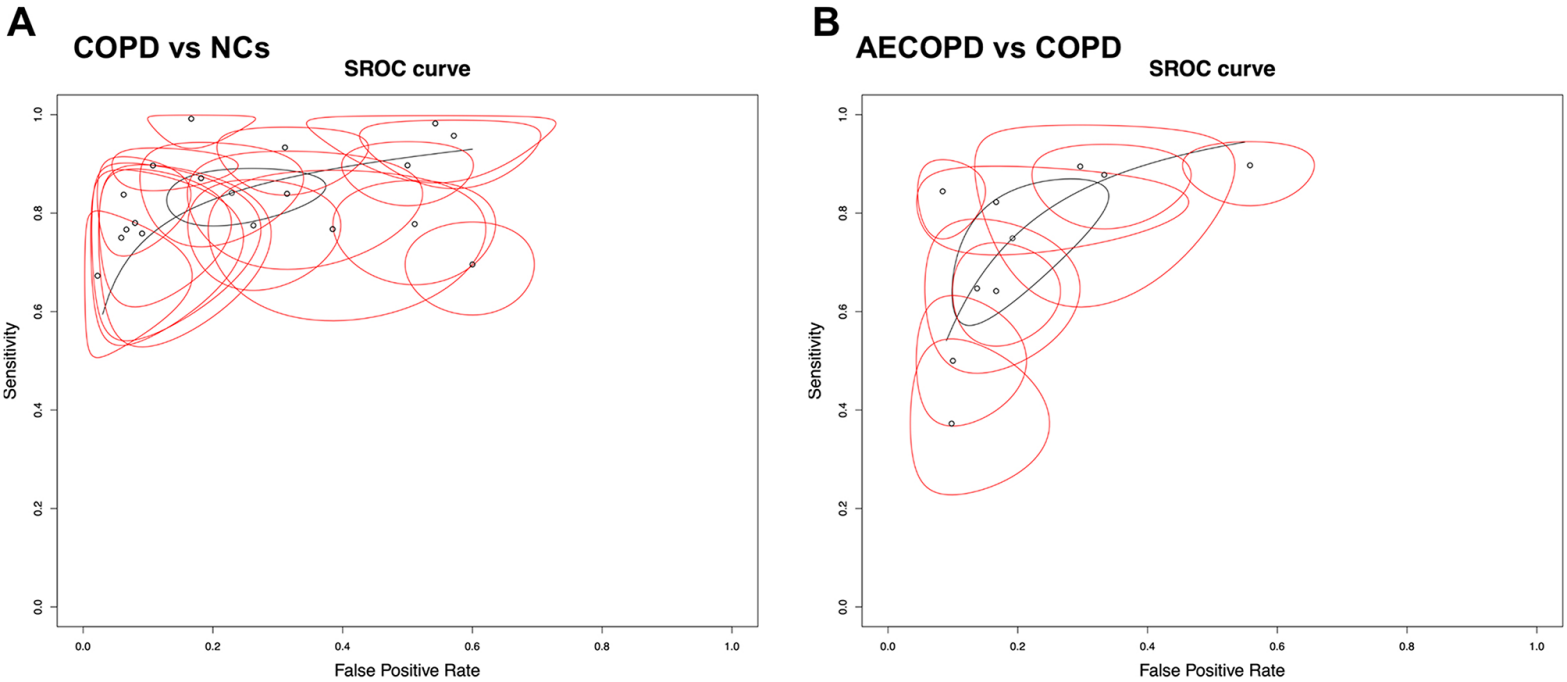
SROC of lncRNAs for the diagnosis in COPD. **(A)** COPD vs. NCs, **(B)** AECOPD vs. COPD. AECOPD, acute exacerbations of Chronic obstructive pulmonary disease. COPD, Chronic obstructive pulmonary disease. NC, normal control

A total of 17 literature [13–15, 25–38] were eligible for inclusion in the meta-analysis of diagnostic performance. All the included literature were published from 2019 to 2023 and conducted in China, with a total of 2983 participants involved. There were 7 literature [13, 14, 25, 26, 29, 35, 36] that included AECOPD patients, stable COPD patients, and normal controls (NCs), 7 literature [27, 28, 31–33, 37, 38] that recruited COPD patients and NCs, and 3 literature [15, 30, 34] that compared AECOPD patients with COPD patients. The specimen sources were mainly plasma, serum, peripheral blood mononuclear cells (PBMCs), and lung tissue. All 15 lncRNAs involved, 10 lncRNAs were up-regulated (*NEAT1*, *NR-026690*, *ENST00000447867*, *PACER*, *MALAT1*, *PVT1*, *OIP5-AS1*, *LUCAT1*, *MEG3*, *LINC00599*) [13–15, 26, 27, 29, 31, 33, 35–37] and 5 were down-regulated (*ANRIL*, *LINC00987*, *Lnc-IL7R*, *CASC2*, *LINC00612*) [25, 28, 30, 32, 34, 38]. The characteristics of the included literature were summarized in Tables 1 and 2. The QUADAS-2 assessment indicated that studies were at moderate to high risk of bias and mostly had concerns about patient selection, as shown in Supplementary Figure S1. In order to improve the diagnostic test accuracy, we mainly extracted TP, FP, FN, TN, SEN, SPE, and AUC from the included literature [13–15, 25–38]. First, we enrolled 18 terms [13, 14, 25–29, 31–33, 35–38] to report the diagnostic role of lncRNA in comparison to COPD patients and NCs. The SEN and SPE of the diagnostic accuracy for lncRNA in COPD and NCs were 0.86 (95% confidence interval (CI) [0.80, 0.90]) and 0.78 (95% CI [0.67, 0.86]), respectively. The DOR was 21.09 (95% CI [11.39, 40.91]), and forest plots of that were seen in Fig. 3A. It was worth reminding that when comparing stable COPD patients with NCs, the SEN, SPE, and DOR were 0.80 (95% CI [0.75, 0.85]), 0.81 (95% CI [0.67, 0.90]), and 16.87 (95% CI [7.77, 36.65]), respectively. Subsequently, the SEN, SPE, and DOR were 0.93 (95% CI [0.86, 0.97]), 0.72 (95% CI [0.54, 0.85]), and 35.98 (95% CI [11.70, 110.66]) comparing AECOPD and NCs. AECOPD is an acute event characterized by a worsening of respiratory symptoms that requires a change in therapy. Also, we reported the SEN, SPE, and DOR between AECOPD and COPD (Fig. 3B); the results showed 0.75 (95% CI [0.62, 0.85]), 0.81 (95% CI [0.71, 0.89]), and 13.02 (95% CI [7.76, 21.85]). Meanwhile, we plotted the sROC curve and calculated the AUC to assess diagnostic accuracy, as shown in Fig. 4, suggesting a certain diagnostic accuracy of overall lncRNAs in distinguishing COPD patients and NCs (AUC = 0.876) and a higher diagnostic accuracy in detecting AECOPD patients and COPD (AUC = 0.847).

**Table 1:** Characteristics of literature enrolled

Study	Patients (controls)	Specimen	Method	LncRNA(Regulated)
Ge2019 [25]	AECOPD/COPD/NC(136/138/140)	Plasma	qRT-PCR	*ANRIL*(D)
Ming2019 [13]	AECOPD/COPD/NC(90/90/90)	Plasma	qPCR	*NEAT1*(U)
Qi2019^†^ [26]	AECOPD/COPD/NC(56/56/35)	PBMCs	qRT-PCR	*NR-026690*(U)
Qi2019^†^ [26]	AECOPD/COPD/NC(56/56/35)	PBMCs	qRT-PCR	*ENST00000447867*(U)
Du2020 [27]	COPD/NC(55/45)	Serum	qRT-PCR	*PACER*(U)
Liu2020 [14]	AECOPD/COPD/NC(120/120/120)	Plasma	RT-qPCR	*MALAT1*(U)
Wang2020a [28]	COPD/NC(29/33)	Lung tissue	RT-qPCR	*LINC00987*(D)
Wang2020b [29]	AECOPD/COPD/NC(80/80/80)	PBMCs	RT-qPCR	*PVT1*(U)
Wu2020 [30]	AECOPD/COPD(19/27)	Plasma	qRT-PCR	*Lnc-IL7R*(D)
Chen2021 [15]	AECOPD/COPD(122/178)	Plasma	qRT-PCR	*NEAT1*(U)
Hao2021 [31]	COPD/NC(62/55)	Blood	qRT-PCR	*OIP5-AS1*(U)
Liu2021 [32]	COPD/NC(50/50)	Serum	qRT-PCR	*CASC2*(D)
Zhao2021 [33]	COPD/NC(70/72)	Blood	qRT-PCR	*LUCAT1*(U)
Bamodu2022 [34]	AECOPD/COPD(18/97)	Plasma	qRT-PCR	*Lnc-IL7R*(D)
Mao2022 [35]	AECOPD/COPD/NC(135/165/65)	Serum	qRT-PCR	*PVT1*(U)
Wei2022 [36]	AECOPD/COPD/NC(28/15/26)	Blood	qRT-PCR	*MEG3*(U)
Xu2022 [37]	COPD/NC(30/30)	Lung tissue	RT-qPCR	*LINC00599*(U)
Xiao2023 [38]	COPD/NC(32/34)	Blood	RT-qPCR	*LINC00612*(D)

**Table 2:** Diagnosis performance of enrolled literature in the meta-analysis

Study	lncRNA	TP	FP	FN	TN	SEN%	SPE%	AUC
COPD vs. NC								
Ge2019 [25]	*ANRIL*	96	84	42	56	69.6%	40.0%	0.543
Ming2019 [13]	*NEAT1*	70	46	20	44	77.8%	48.9%	0.642
Du2020 [27]	*PACER*	37	1	18	44	66.7%	97.8%	0.867
Wang2020a [28]	*LINC00987*	22	3	7	30	76.2%	91.2%	0.798
Wang2020b [29]	*PVT1*	62	21	18	59	77.6%	73.9%	0.813
Hao2021 [31]	*OIP5-AS1*	54	10	8	45	87.1%	81.4%	0.903
Liu2021 [32]	*CASC2*	39	4	11	46	78.0%	92.0%	0.924
Zhao2021 [33]	*LUCAT1*	67	40	3	30	95.5%	42.4%	0.892
Mao2022 [35]	*PVT1*	269	7	31	58	89.6%	89.7%	0.878
Wei2022 [36]	*MEG3*	33	10	10	16	76.9%	60.0%	0.661
Xu2022 [37]	*LINC00599*	23	2	7	28	76.7%	93.3%	0.896
Xiao2023 [38]	*LINC00612*	24	2	8	32	75.0%	93.9%	0.929
AECOPD vs. NC								
Ge2019 [25]	*ANRIL*	122	70	14	70	89.7%	50.0%	0.700
Ming2019 [13]	*NEAT1*	84	28	6	62	93.3%	68.9%	0.869
Qi2019^†^ [26]	*NR-026690*	55	19	1	16	98.2%	46.4%	0.757
Qi2019^†^ [26]	*ENST00000447867*	47	11	9	24	83.3%	68.2%	0.721
Liu2020 [14]	*MALAT1*	119	20	1	100	99.2%	83.3%	0.969
Wang2020b [29]	*PVT1*	67	5	13	75	83.6%	93.8%	0.953
AECOPD vs. COPD								
Ge2019 [25]	*ANRIL*	122	77	14	61	89.7%	44.2%	0.659
Ming2019 [13]	*NEAT1*	79	28	11	56	87.8%	62.2%	0.779
Qi2019^†^ [26]	*NR-026690*	33	7	18	44	64.3%	86.0%	0.838
Qi2019^†^ [26]	*ENST00000447867*	19	5	32	46	38.1%	89.3%	0.666
Liu2020 [14]	*MALAT1*	77	20	43	100	64.2%	83.3%	0.846
Wang2020b [29]	*PVT1*	40	8	40	72	50.0%	90.0%	0.739
Wu2020 [30]	*Lnc-IL7R*	17	8	2	19	89.5%	70.4%	0.856
Chen2021 [15]	*NEAT1*	103	15	19	163	84.4%	91.6%	0.887
Bamodu2022 [34]	*Lnc-IL7R*	88	3	19	15	83.0%	83.3%	0.858

### Subgroup analysis and meta-regression analysis

To explore the potential sources of heterogeneity in the enrolled literature, we continued to conduct a series of analyses, mainly subgroup analysis and meta-regression analysis. There was a lot of uncertain heterogeneity, including the types of specimens and dysregulated lncRNA in this study. Therefore, we plotted a forest plot of the SEN, the SPE, and the DOR based on the differences in the types of samples and dysregulated lncRNA, as shown in Supplementary Figure S2, S3. Taken together, differential types of samples and dysregulated lncRNA did not influence the result of diagnostic efficiency, but there were differences in that. For further investigation of the mixed relations listed before, we performed meta-regression analysis based on the types of specimens and dysregulated lncRNA (Table 3). It was found that the SEN, SPE, and DOR were influenced by different specimens and regulated by lncRNA. Next, it showed that there were differences (*P* < 0.05) in multiple Meta-regressions of differential specimens and dys-regulated lncRNA except for SEN for distinguishing AECOPD and COPD (*P* = 0.51). Simultaneously, we found the *NEAT1* [13, 15], *PVT1* [29, 35] and *Lnc-IL7R* [30, 34] had been studied by a number of scholars, they had significant diagnostic efficiency (*P* < 0.05), seen in Supplementary Figure S4.

**Table 3:** Assessment of meta-regression analysis (z-value(P-value))

study	SEN		SPE		DOR	
	Specimen	Regulated	Specimen	Regulated	Specimen	Regulated
COPD vs. NCs	6.31(< 0.01)	3.99(< 0.01)	2.73(< 0.01)	2.60(< 0.01)	7.40(< 0.01)	3.77(< 0.01)
AECOPD vs. NCs	4.15(< 0.01)	3.50(< 0.01)	1.95(0.05)	1.88(0.06)	3.74(< 0.01)	3.05(< 0.01)
AECOPD vs. COPD	0.09(0.93)	3.03(< 0.01)	4.53(< 0.01)	2.49(0.01)	4.61(< 0.01)	5.62(< 0.01)

### Sensitivity analysis and publication bias

**Fig. 5:**
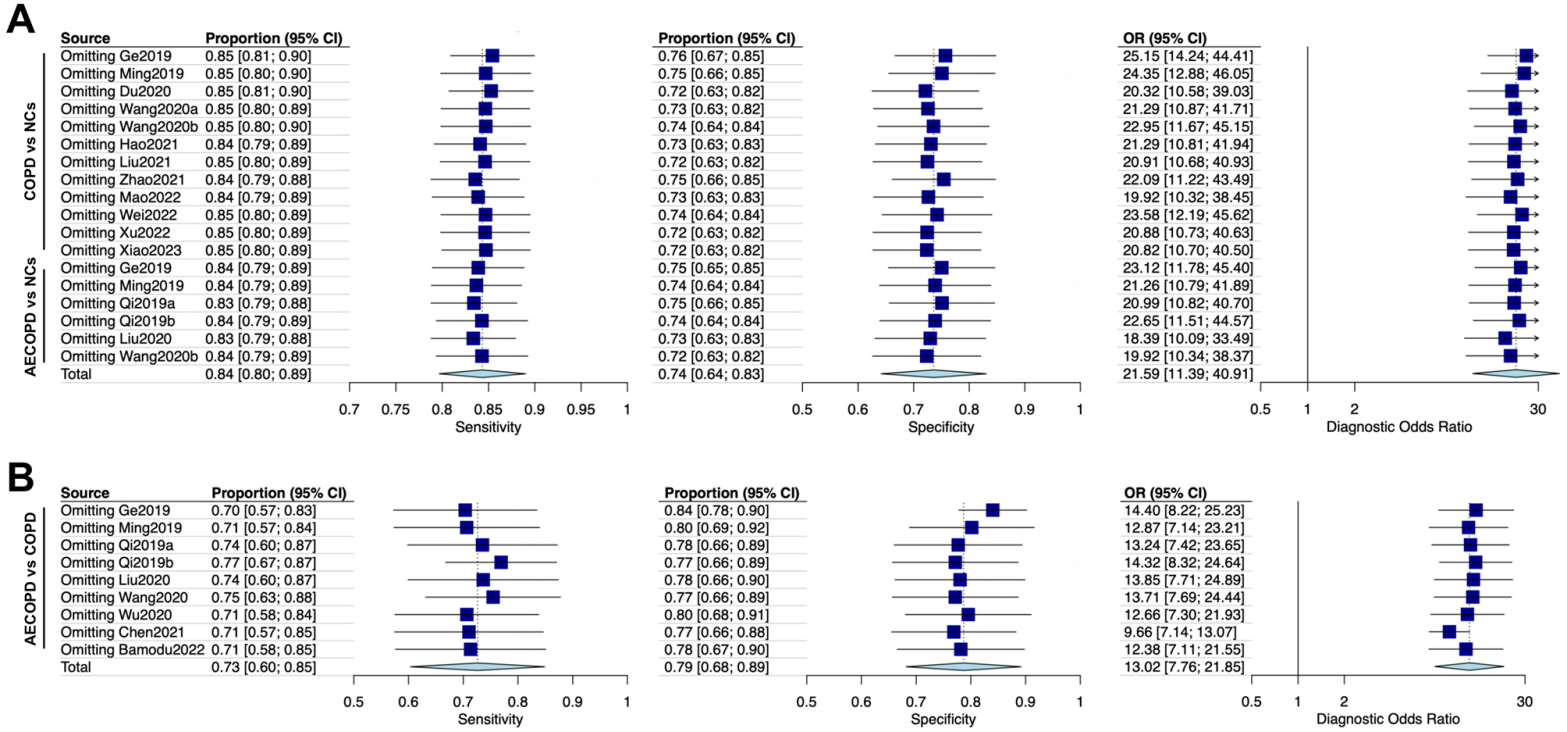
Sensitivity analysis of the result of this meta-analysis. **(A)** COPD vs. NCs, **(B)** AECOPD vs. COPD. AECOPD, acute exacerbations of Chronic obstructive pulmonary disease. COPD, Chronic obstructive pulmonary disease. NC, normal control

**Fig. 6:**
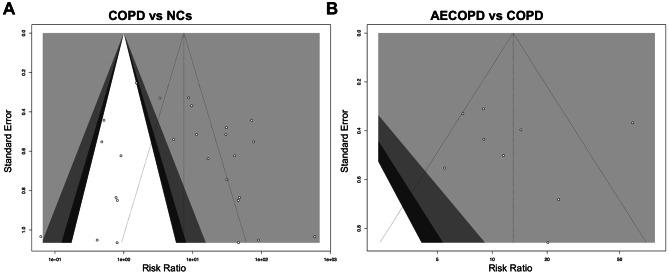
Funnel plot of publication bias for diagnostic accuracy of COPD. **(A)** COPD vs. NCs, **(B)** AECOPD vs. COPD. AECOPD, acute exacerbations of Chronic obstructive pulmonary disease. COPD, Chronic obstructive pulmonary disease. NC, normal control

After the contribution of each study to the pooled values had been assessed by sensitivity analysis, we reappraised the pooled estimates by excluding each study one at a time and calculating for the remaining studies. Recalculating pooled values for the SEN, SPE, and DOR, the result indicated that excluding any one literature did not cause a change in the pooled estimates in the result of meta-analysis, as shown in Fig. 5. Also, when we plotted the funnel plot of the included literature, it suggested that there was a certain publication bias in the diagnostic accuracy of COPD and AECOPD with NCs, and the publication bias was viewed (Fig. 6).

### Biological function of identified lncRNA

**Fig. 7:**
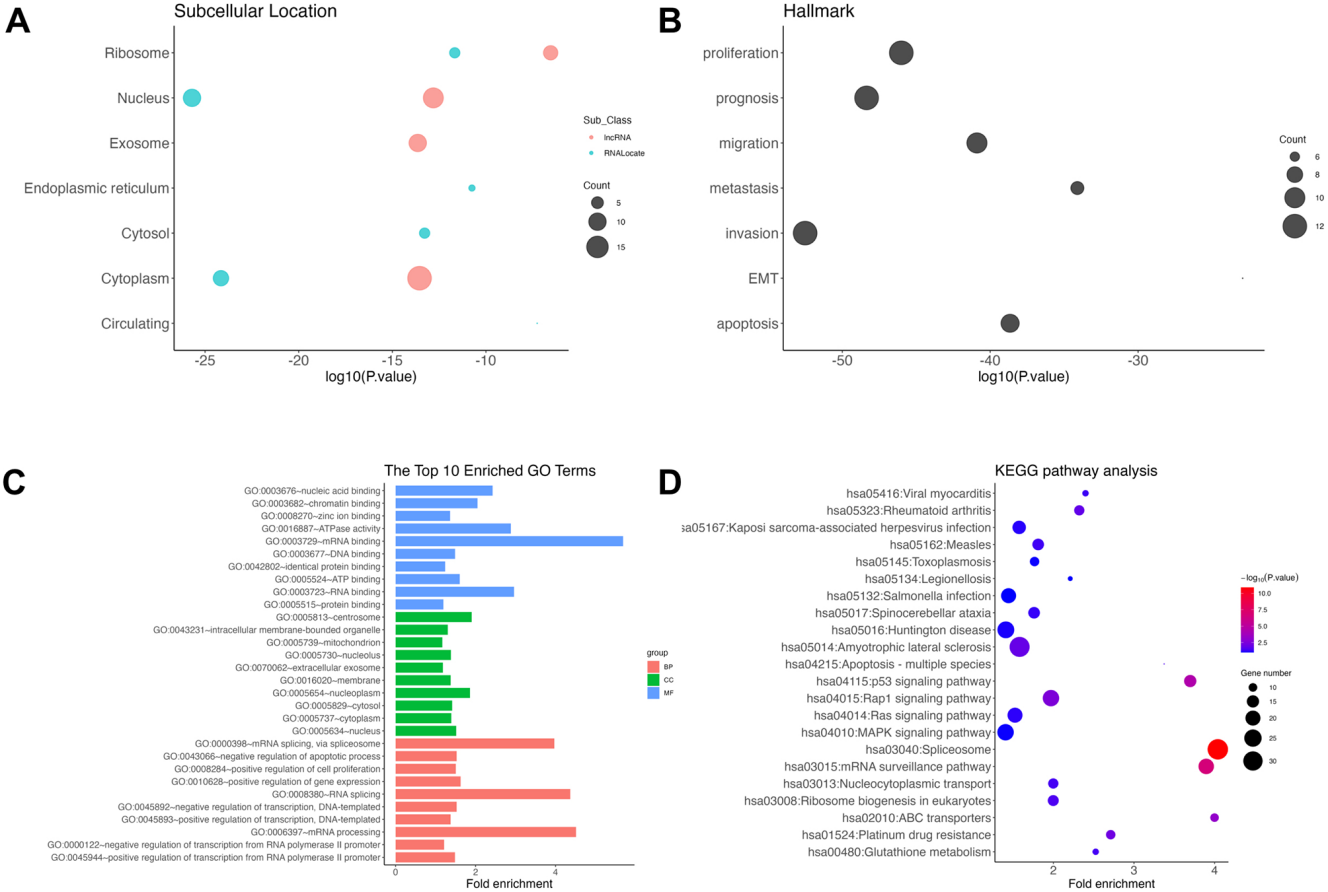
Bioinformatic analysis of identified lncRNAs. **(A)** The subcellular location of identified lncRNAs, **(B)** The hallmark of identified lncRNAs, **(C)** GO enrichment analysis, B. KEGG pathways analysis. GO, Gene ontology. Kyoto encyclopedia of genes and genomes, KEGG

We aimed to find the potential biological function; the subcellular location, hallmark, and lncRNA binding protein were searched on the LncSEA platform. As shown in Fig. 7, the identified lncRNAs were mainly located in the nucleus and cytoplasm, and they were involved in the pathways of proliferation, invasion, and prognosis. Also, GO enrichment and KEGG pathway analysis were made, and the molecular function (MF) was concentrated on mRNA binding, RNA binding, nucleic acid binding, etc. The cellular component (CC) was located in the nucleoplasm, centrosome, nucleus, etc. mRNA processing, RNA splicing, and mRNA splicing via spliceosom were mainly biological process (BP). The results of KEGG pathway analysis showed that the lncRNA binding protein was involved in the spliceosome, Rap1 signaling pathway, MAPK signaling pathway, etc.

## Discussion

Globally, COPD has become the highest morbidity and mortality chronic pulmonary disease [1, 2, 39]. Early diagnosis and intervention in COPD can help prevent and control disease progression effectively and improve the quality of life of patients significantly. However, approximately 70–80% of adults with COPD might be underdiagnosed early [6, 8, 40]. In clinical practice, adults with undiagnosed COPD have fewer respiratory symptoms (e.g., cough, wheezing, and chest pain) than those that have been diagnosed with COPD [41], causing difficulties to detect and diagnose. Individuals with a mild stage might be inclined to ignore the symptoms and not consult a doctor. Furthermore, COPD patients might have clinical symptoms covered up by drugs. Spirometry is used for the diagnosis of COPD and the staging of its severity [42, 43]. Individuals with serious symptoms usually find it hard to complete testing of pulmonary function, which complicates the diagnosis. On the other hand, there are a lot of patients subjected to misdiagnosis at the primary hospital because of a lack of spirometry use [8]. Therefore, it needs to look for new biomarkers to help us diagnose COPD early and correctly.

As an epigenetic regulatory molecule, lncRNA is widely involved in the occurrence and development of COPD. We found a gradual increase in the amount of literature on the role of lncRNAs in COPD since 2000, especially since the growth rate increased significantly after 2016. These findings revealed that most authors have focused on the role of lncRNA in the diagnosis and treatment of COPD. There are a lot of literature [11, 17, 44] suggesting that lncRNA plays a vital role in regulating chronic lung diseases. However, the diagnostic value of lncRNA in COPD is not clear, and the relationship between the alterations of lncRNA and the severity of COPD has to be further studied. This is the first meta-analysis on the diagnostic accuracy of lncRNAs for COPD. Our meta-analysis had enrolled 17 literature [13–15, 25–38] through searching multiple electron databases, there are 10 upregulated [13–15, 26, 27, 29, 31, 33, 35–37] and 5 downregulated [25, 28, 30, 32, 34, 38]. COPD patients might experience two clinical conditions: stable status and AECOPD [3, 45]. Most of the enrolled literature had analyzed expression of lncRNAs in different clinical statuses of COPD, and the AUC of the sROC curve was above 0.8, suggesting excellent diagnostic accuracy for differentiating COPD patients from NCs as well as distinguishing AECOPD and COPD.

We also reviewed the sources of heterogeneity in the enrolled literature. Types of specimens, dysregulated lncRNA, and detection methods were likely to be associated with the interfering factor of lncRNA in the diagnosis of COPD. There had been a number of differences in the SEN, SPE, and DOR in the meta-analysis. Although the results did not affect the diagnostic efficacy, venous blood might be a more convenient clinical testing sample. Generally, the purity of RNA extracted from PBMCs is higher than that from blood, but the extraction process of PBMCs is complicated and time-consuming relatively [46]. Furthermore, lncRNAs might be detected by different PCR methods, which should pass strict quality control in a suitable laboratory. On the other part, due to the spatiotemporal characteristics of lncRNAs, it is a challenge for the collection and preservation of clinical specimens.

This study illustrated that both the up-regulated and down-regulated lncRNAs appeared to have similar diagnostic accuracy. In clinical practice, high expression of lncRNAs is easy to detect. Hence, we advise that up-regulated lncRNAs should be a better choice for diagnosis accuracy. *NEAT1* is a lncRNA found to be expressed widely in a variety of mammalian cell types and involved in the occurrence and development of a variety of diseases [47, 48]. Ming et al. found high expression of *NEAT1* in AECOPD and COPD patients; the increased expression of *NEAT1* may be associated with the stage of COPD and positively correlated with GOLD grade [13, 15], and the underlining mechanisms were considered to regulate inflammation. Likewise, *MALAT1* was overexpressed in COPD lung tissue specimens [49], and was positively correlated with GOLD grade in both AECOPD and COPD patients; it is believed that MALAT1 might have a relationship with acute exacerbation risk of COPD [14]. It was believed that *PVT1* combined with miR-146a induced the change of pulmonary function in COPD smokers [50], *PVT1* should have the ability to predict COPD susceptibility and acute exacerbation risk and be correlated with GOLD stage and inflammatory cytokine levels in AECOPD patients and stable COPD patients [29, 35]. It was suggested that lncRNA may represent a potent diagnostic and therapeutic biomarker in COPD.

LncRNAs were often considered to play critical roles in various cellular functions, occurrence and development of multiple diseases [10, 51, 52]. We identified these lncRNAs that had been certified to regulate cellular processes such as mRNA binding, spliceosomes, and nucleic acid binding, which would drive more researchers to explore how lncRNAs influence the development and occurrence of COPD. According to their location in the cell relative to protein-coding genes, they can be classified as sense, antisense, intronic, intergenic, and enhancer lncRNAs. *PTPRE-AS1* [53] and *TTN-AS1* [54] played a positive role in inflammation disease as an antisense lncRNA. The mechanisms by which the biological function of lncRNAs is regulated are rather complicated and have not yet been fully elucidated. We employed the lncRNA binding protein to investigate the potential biological processes. The results of the KEGG pathways analysis showed the Rap1 signaling pathway and the MAPK signaling pathway, which are involved in inflammation and apoptosis. It is believed that p38 MAPK plays a key role in signaling mechanisms underlying COPD pathobiology [55, 56], and there are some new natural compounds for treating COPD that are mainly attained through MAPK inflammatory signaling pathways and Nrf2 oxidative stress signaling pathways [57]. It was suggested that lncRNAs might participate in the regulation of inflammatory pathways and thus affect the occurrence and development of COPD.

Also, there were some limitations in this study. First, despite the fact that the quality assessment of the included literature was relatively reliable through the QUADAS-2 checklist, the included literature and the sample size were relatively small, which might have induced possible heterogeneity. Second, there is a large publication bias; we considered that the authors of enrolled literature might be apt to submit positive results, and we may need more clinical data to get the results closer to the truth. Third, all the included literature [13–15, 25–38] was domestic. As is known to all, there are genetic differences between different races. LncRNAs from the enrolled literature were appropriate for Chinese COPD patients, it is not surprising that different races might have different genetic expression and lead to different results.

## Conclusion

In conclusion, this study indicated that lncRNAs could serve as diagnostic biomarkers and therapeutic targets for COPD. LncRNAs often show significant differential expression between COPD patients and healthy people, and have the ability to differentiate AECOPD from COPD patients. In mechanism, identified lncRNAs might be involved in molecular biological function-regulated occurrence and development of COPD. Future studies should be designed to overcome the major limitations shown by the differences in lncRNA estimates and detection methods. Moreover, a number of lncRNAs from the current evidence base, such as *NEAT1*, *MALAT1*, and *PVT1*, should be further studied to verify their value in diagnosis and therapy through randomized controlled trials (RCT), which might involve larger patient samples.

### Electronic supplementary material

Below is the link to the electronic supplementary material.

Supplementary Material 1

Supplementary Material 2

Supplementary Material 3

Supplementary Material 4

Supplementary Material 5

Supplementary Material 6

Supplementary Material 7

Supplementary Material 8

Supplementary Material 9

Supplementary Material 10

Supplementary Material 11

## Data Availability

All data generated or analysed during this study are included in this published article and its supplementary materials. The R script for this study is openly available in github.com at https://raw.githubusercontent.com/linqitc/MyRScript/master/Meta-analysis_LncRNA.r.

## Abbreviations

AECOPD: Acute exacerbation of chronic obstructive pulmonary disease
AUC: Area under curve
BP: Biological process
CI: Confidence interval
CC: Cellular component
CENTRAL: Cochrane Central Register of Controlled Trials
CNKI: China National Knowledge Infrastructure
COPD: Chronic obstructive pulmonary disease
DOR: Diagnostic odds ratio
DTA: Diagnostic test accuracy
FN: False negative
FP: False positive
GO: Gene ontology
GOLD: The Global Initiative for Chronic Obstructive Lung Disease
LncRNA: Long non-coding RNA
KEGG: Kyoto encyclopedia of genes and genomes
MF: Molecular function
NC: Normal control
PBMC: Peripheral blood mononuclear cell
PROSPERO: The International Prospective Register of Systematic Reviews
PRISMA: Preferred Reporting Items for Systematic reviews and Meta-Analyses
QUADAS-2: Quality Assessment of Diagnostic Accuracy Studies 2
RCT: Randomized controlled trials
SEN: Sensitivity
SinoMed: China Biology Medicine
SPE: Specificity
sROC: Summary receiver operating characteristic curve
TP: True positive
TN: True negative
WOS: Web of Science
