# Supplementary figures and images for: The utility of long non-coding RNAs in chronic obstructive pulmonary disease: a comprehensive analysis
Source: BMC Pulm Med. 2023 Sep 11;23:340. doi: 10.1186/s12890-023-02635-w (PMC10496340; doi:10.1186/s12890-023-02635-w)

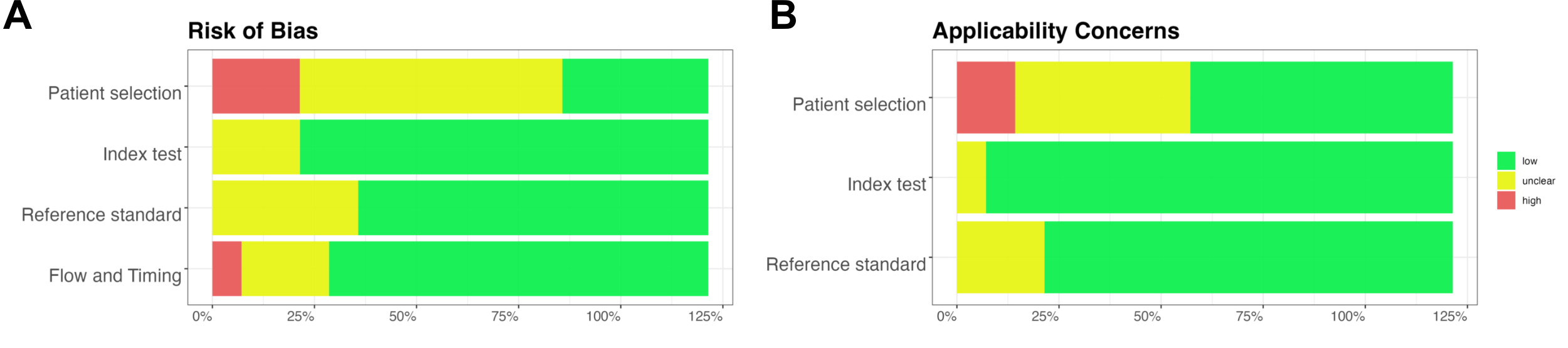

Supplement: Supplementary file 1 — Supplementary Material 1 [file 12890_2023_2635_MOESM1_ESM.jpg]

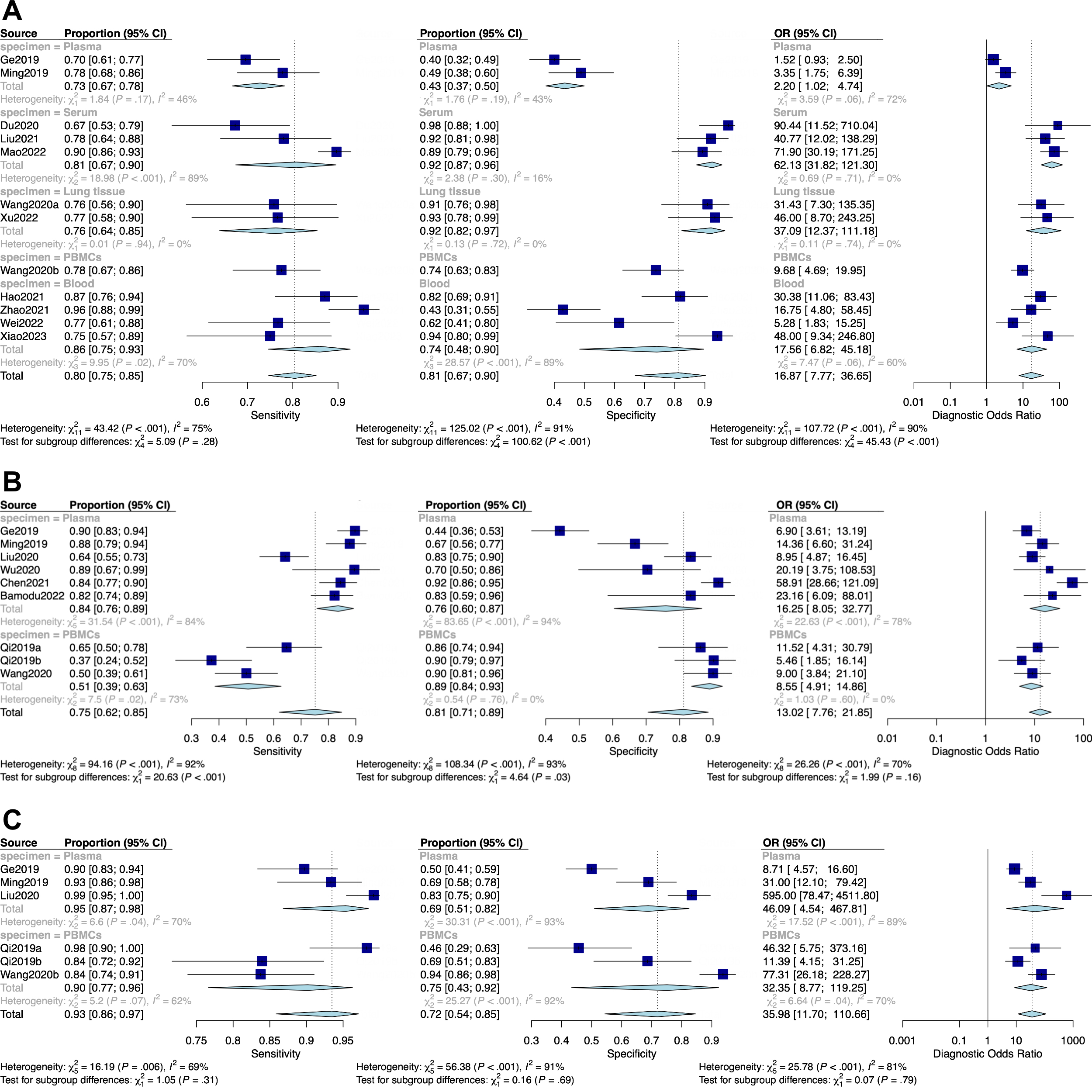

Supplement: Supplementary file 2 — Supplementary Material 2 [file 12890_2023_2635_MOESM2_ESM.jpg]

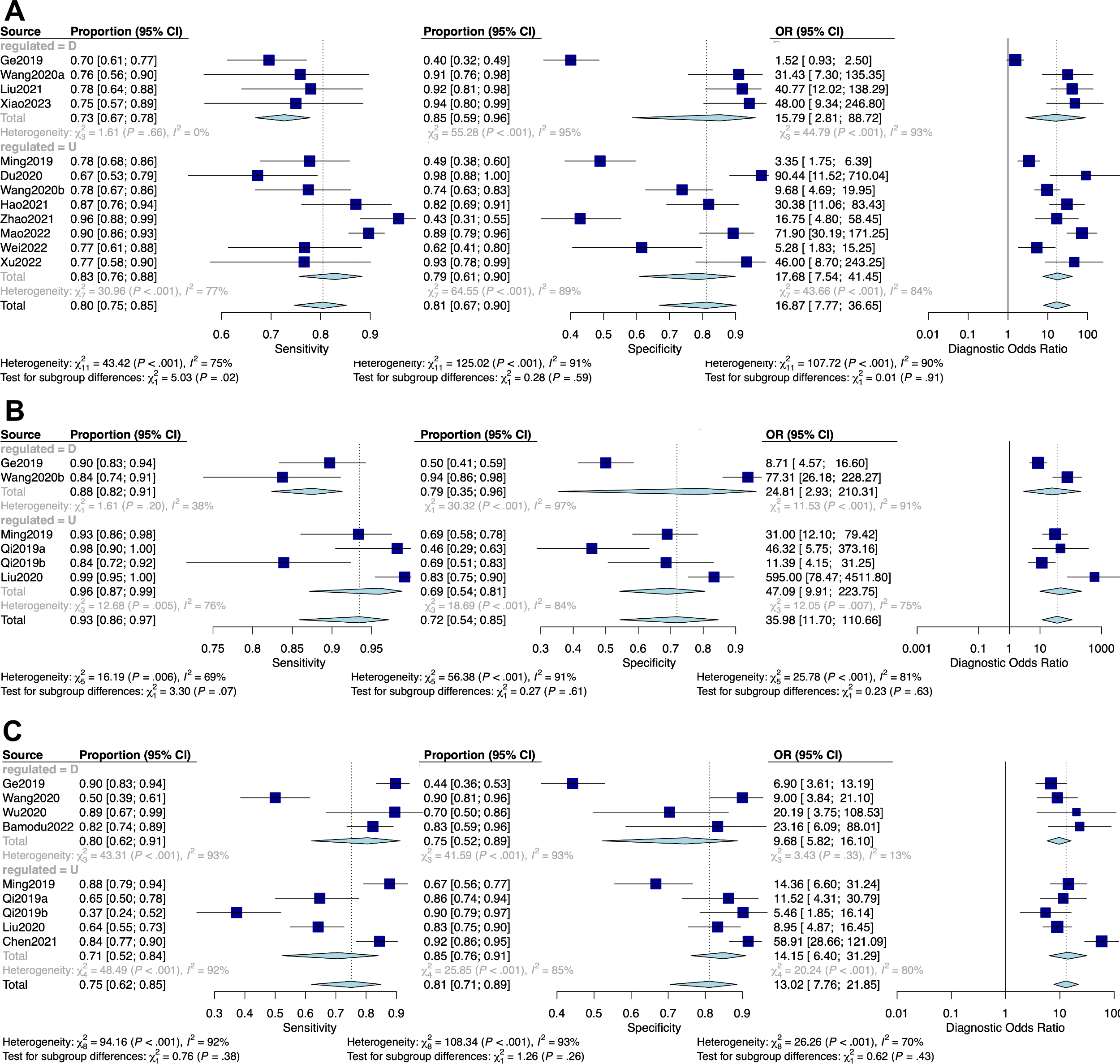

Supplement: Supplementary file 3 — Supplementary Material 3 [file 12890_2023_2635_MOESM3_ESM.jpg]

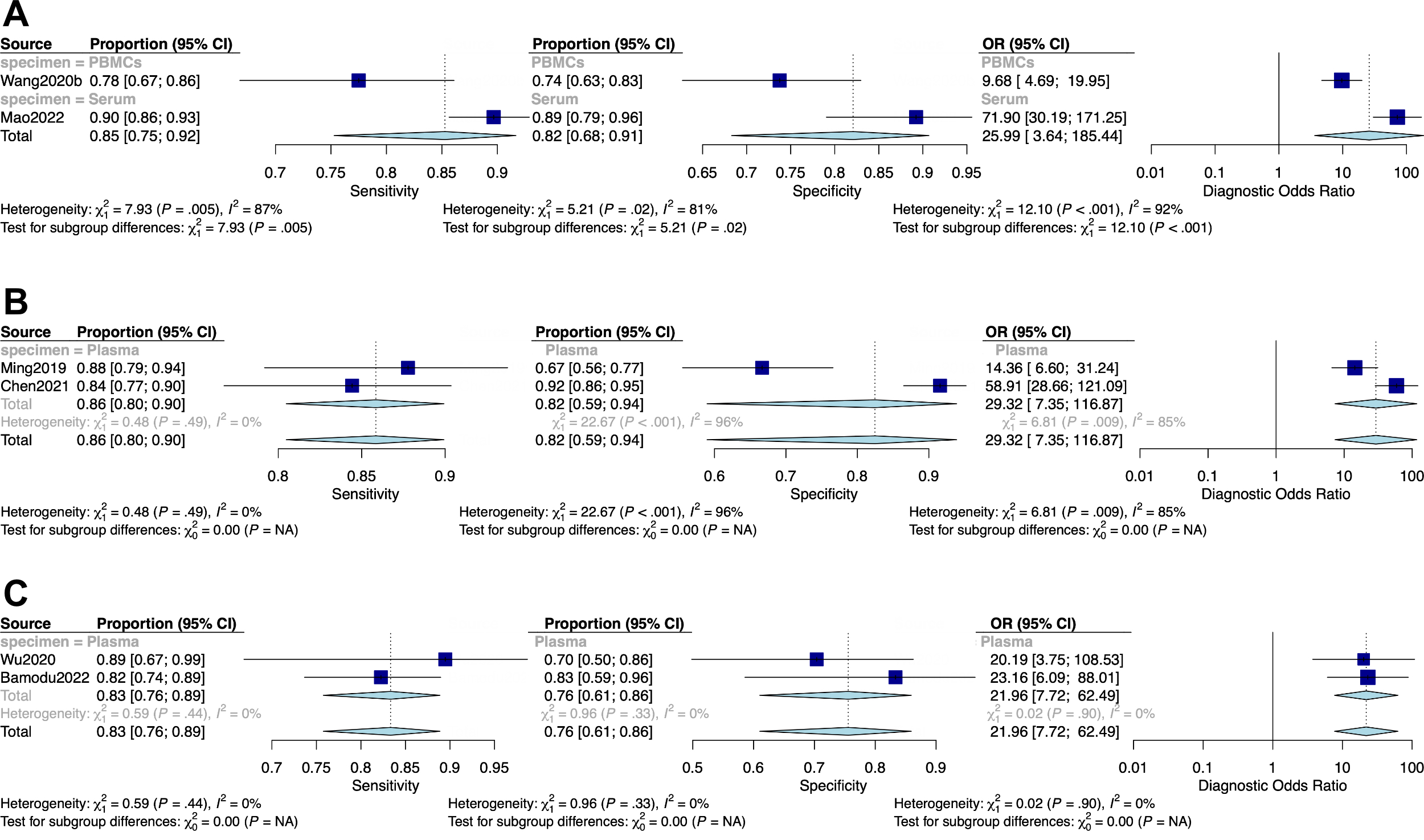

Supplement: Supplementary file 4 — Supplementary Material 4 [file 12890_2023_2635_MOESM4_ESM.jpg]
