# Supplementary material for: The utility of long non-coding RNAs in chronic obstructive pulmonary disease: a comprehensive analysis
Source: BMC Pulm Med. 2023 Sep 11;23:340. doi: 10.1186/s12890-023-02635-w (PMC10496340; doi:10.1186/s12890-023-02635-w)
Supplement: Supplementary file 5 — Supplementary Material 5 [file 12890_2023_2635_MOESM5_ESM.doc]

| # | Search Query | Results |
| --- | --- | --- |
| 1 | ALL = (Pulmonary Disease, Chronic Obstructive) OR ALL = (Lung Diseases, Obstructive) OR ALL = (emphysema*) OR ALL = (Airflow Obstruction, Chronic) OR ALL = (Chronic Airflow Obstruction) OR ALL = (Chronic Obstructive Airway Disease) OR ALL = (Chronic Obstructive Pulmonary Disease) OR ALL = (Chronic Obstructive Lung Disease) OR ALL = (COPD) OR ALL = (COAD) OR ALL = (cold) OR ALL = (aect) | 588254 |
| 2 | ALL = (long untranslated rna) OR ALL = (long noncoding rna) OR ALL = (long non-coding rna) OR ALL = (long intergenic non-protein coding rna) OR ALL = (long non-protein-coding rna) OR ALL = (long ncrna) OR ALL = (long ncrnas) OR ALL = (lncrna) OR ALL = (lincrna) | 57311 |
| 3 | #2 AND #1 | 660 |

Table S1 WOS Search strategy
