# Supplementary material for: The utility of long non-coding RNAs in chronic obstructive pulmonary disease: a comprehensive analysis
Source: BMC Pulm Med. 2023 Sep 11;23:340. doi: 10.1186/s12890-023-02635-w (PMC10496340; doi:10.1186/s12890-023-02635-w)
Supplement: Supplementary file 6 — Supplementary Material 6 [file 12890_2023_2635_MOESM6_ESM.doc]

Table S2 Pubmed search strategy

| Search number | Query | Results |
| --- | --- | --- |
| 1 | (Pulmonary Disease, Chronic Obstructive[MeSH Terms]) AND (Pulmonary Disease, Chronic Obstructive[MeSH Terms]) | 66,951 |
| 2 | Lung Diseases, Obstructive[MeSH Terms] | 236,972 |
| 3 | emphysema* | 39,803 |
| 4 | COPD OR COAD OR COBD OR AECB | 108,160 |
| 5 | Airflow Obstruction, Chronic | 98,915 |
| 6 | Chronic Airflow Obstruction | 98,915 |
| 7 | Chronic Obstructive Airway Disease | 98,934 |
| 8 | Chronic Obstructive Lung Disease | 101,026 |
| 9 | Chronic Obstructive Pulmonary Disease | 97,808 |
| 10 | #1 OR #2 OR #3 OR #4 OR #5 OR #6 OR #7 OR #8 OR #9 | 288,300 |
| 11 | RNA, Long Noncoding[MeSH Terms] | 32,831 |
| 12 | LincRNAs OR lncRNA | 52,517 |
| 13 | LINC RNA | 46,522 |
| 14 | Long ncRNA OR Long ncRNAs | 46,695 |
| 15 | Long Noncoding RNA OR Long Non-Coding RNA | 47,600 |
| 16 | Long Intergenic Non-Protein Coding RNA | 46,500 |
| 17 | Long Non-Protein-Coding RNA | 47,153 |
| 18 | RNA, Long Non-Translated OR RNA, Long Untranslated | 49,017 |
| 19 | #11 OR #12 OR #13 OR #14 OR #15 OR #16 OR #17 OR #18 | 56,132 |
| 20 | #10 and #19 | 353 |
