# Supplementary material for: The utility of long non-coding RNAs in chronic obstructive pulmonary disease: a comprehensive analysis
Source: BMC Pulm Med. 2023 Sep 11;23:340. doi: 10.1186/s12890-023-02635-w (PMC10496340; doi:10.1186/s12890-023-02635-w)
Supplement: Supplementary file 7 — Supplementary Material 7 [file 12890_2023_2635_MOESM7_ESM.doc]

Table S3 Embase search strategy

| No. | Query | Results |
| --- | --- | --- |
| 1 | 'chronic obstructive pulmonary disease'/exp OR 'chronic obstructive pulmonary disease' | 185806 |
| 2 | 'emphysema':ab,ti,kw | 39621 |
| 3 | 'chronic obstructive lung disease':ti,ab,kw | 6896 |
| 4 | 'chronic obstructive lung disease'/exp | 175833 |
| 5 | 'chronic airflow obstruction':ti,ab,kw OR 'chronic obstructive airway disease':ti,ab,kw | 1272 |
| 6 | 1 or 2 or 3 or 4 or 5 | 216681 |
| 7 | 'long untranslated rna'/exp | 52639 |
| 8 | 'long noncoding rna':ti,ab,kw OR 'long non-coding rna':ti,ab,kw | 25510 |
| 9 | 'long intergenic non-protein coding rna':ti,ab,kw OR 'long non-protein-coding rna':ti,ab,kw | 432 |
| 10 | 'long ncrna':ti,ab,kw OR 'long ncrnas':ti,ab,kw OR 'lncrna':ti,ab,kw OR 'lincrna':ti,ab,kw | 36749 |
| 11 | 7 or 8 or 9 or 10 | 57686 |
| 12 | 6 and 11 | 226 |
