# Supplementary material for: The utility of long non-coding RNAs in chronic obstructive pulmonary disease: a comprehensive analysis
Source: BMC Pulm Med. 2023 Sep 11;23:340. doi: 10.1186/s12890-023-02635-w (PMC10496340; doi:10.1186/s12890-023-02635-w)
Supplement: Supplementary file 8 — Supplementary Material 8 [file 12890_2023_2635_MOESM8_ESM.doc]

Table S4 CENTRAL search strategy

| ID | Search | Results |
| --- | --- | --- |
| 1 | MeSH descriptor Lung Diseases, Obstructive, this term only | 227 |
| 2 | MeSH descriptor Pulmonary Disease, Chronic Obstructive explode all trees | 173 |
| 3 | chronic* near/3 bronchiti* | 2356 |
| 4 | emphysema* | 1954 |
| 5 | (obstruct) near/3 (Pulmonary or lung or airway* or airflow* or bronch* or respirat*) | 123 |
| 6 | (COPD or COAD or COBD or AECB) | 19523 |
| 7 | #1 OR #2 OR #3 OR #4 OR #5 OR #6 | 22539 |
| 8 | MeSH descriptor RNA, Long Noncoding explode all trees | 1 |
| 9 | RNA, Long Non-Translated or RNA, Long Untranslated | 13 |
| 10 | LincRNAs or lncRNA | 103 |
| 11 | LINC RNA | 3 |
| 12 | Long ncRNA or Long ncRNAs | 2 |
| 13 | Long Noncoding RNA or Long Non-Coding RNA | 164 |
| 14 | Long Intergenic Non-Protein Coding RNA | 3 |
| 15 | Long Non-Protein-Coding RNA | 3 |
| 16 | #8 OR #9 OR #10 OR #11 OR #12 OR #13 OR #14 OR #15 | 194 |
| 17 | #7 and #16 | 4 |
