# Supplementary material for: The utility of long non-coding RNAs in chronic obstructive pulmonary disease: a comprehensive analysis
Source: BMC Pulm Med. 2023 Sep 11;23:340. doi: 10.1186/s12890-023-02635-w (PMC10496340; doi:10.1186/s12890-023-02635-w)
Supplement: Supplementary file 9 — Supplementary Material 9 [file 12890_2023_2635_MOESM9_ESM.doc]

Table S5 Chinese electric database search strategy

| Database | Search strategy | result |
| --- | --- | --- |
| CNKI | SU=(‘Chronic obstructive pulmonary disease’ + ‘emphysema’ + ‘Chronic bronchitis’ + ‘COAD’ + ‘COPD’)*(‘long non-coding RNA’ + ‘Long Noncoding RNA’ + ‘LincRNAs’ + ‘lncRNA’ + ‘Long ncRNA’) | 25 |
| WanFang Data | ((long non-coding RNA OR Long Noncoding RNA OR LincRNAs OR lncRNA OR Long ncRNA)) AND ((Chronic obstructive pulmonary disease OR emphysema OR Chronic bronchitis OR COAD OR COPD)) | 38 |
| SinoMed | (‘Chronic obstructive pulmonary disease’ OR ‘emphysema’ OR ‘Chronic bronchitis’ OR ‘COAD’ OR ‘COPD’) AND (‘long non-coding RNA’ OR ‘Long Noncoding RNA’ OR ‘LincRNAs’ OR ‘lncRNA’ OR ‘Long ncRNA’) | 36 |
